# Standardizing the reporting of cholangiocarcinoma: the society of abdominal radiology disease focused panel on cholangiocarinoma lexicon

**DOI:** 10.1007/s00261-024-04769-9

**Published:** 2025-01-07

**Authors:** Robert M. Marks, Hina Arif, Maria Antonietta Bali, Ryan L. Brunsing, Guilherme M. Cunha, Hala Khasawneh, Maria El Homsi, Charanjeet Singh, Raj Paspulati, Andrea Kierans, Aliya Qayyum

**Affiliations:** 1 University of California, San Diego, San Diego, USA; 2 University of Arizona, Tucson, USA; 3 Hopital Universitaire de Bruxelles, Bruxelles, Belgium; 4 Stanford University, Stanford, USA; 5 University of Washington, Seattle, USA; 6 The University of Texas Southwestern Medical Center, Dallas, USA; 7 Memorial Sloan Kettering Cancer Center, New York, USA; 8 Yale University, New Haven, USA; 9 Moffitt Cancer Center, Tampa, USA; 10 Weill Cornell Medical College, New York, USA

**Keywords:** Cholangiocarcinoma, Lexicon, Liver cancer

## Abstract

In March 2023, the Society of Abdominal Radiology (SAR) Disease Focused Panel (DFP) on Cholangiocarcinoma (CCA) was formed. One of its initial tasks was for creation of a lexicon specific for CCA to complement the terms related to the Liver Imaging Reporting and Data System (LI-RADS) category M. A committee was formed and vetted 15 unique terms for CCA. The multidisciplinary members of the DFP passed each term by over 90% approval. The purpose of this paper is to describe the process for developing the lexicon, introduce the lexicon terms, and provide a pictorial atlas of the 15 vetted terms relating to the imaging findings of CCA.

## Introduction

The value of standardized language for reporting imaging findings has been recognized for more than 30 years. Under American College of Radiology (ACR) guidance, the desire for standardized reporting language in mammography led to the development of the breast imaging reporting and data system (BI-RADS) in the early 1990s [1], with many additional “RADS” developed in the years since across different organ systems [2]. For liver imaging, the Liver Imaging Reporting and Data System (LI-RADS) was developed to standardize hepatocellular carcinoma (HCC) diagnosis and treatment response assessment in a select high-risk population, including guidance on image acquisition, interpretation, and reporting [3]. Standardized reporting in LI-RADS aims to provide common language amongst radiologists, clinicians, surgeons, and other health-care professionals to improve clarity of communication amongst stakeholder’s while mitigating inconsistencies and omissions in radiology reports, ultimately improving the diagnosis and treatment of HCC. Standardization also increases patient understanding of imaging results [4].

In LI-RADS, categories are assigned to focal liver observations on imaging to reflect the probability of HCC or other malignancies in patients at risk. The LI-RADS category M describes the imaging features of liver observations that are probably or definitely malignant, however are not specific for HCC, for example cholangiocarcinoma (CCA) or metastatic disease. LI-RADS M imaging features include a mass with targetoid appearance; i.e., rim arterial phase hyperenhancement (APHE), peripheral washout appearance, delayed central enhancement, targetoid diffusion restriction, and/or targetoid transitional or hepatobiliary phase (HBP) signal intensity [5]. For non-targetoid masses, LI-RADS M features include an infiltrative appearance, marked restriction diffusion, necrosis or severe ischemia, or other features suggesting a non-HCC malignancy based on the radiologist assessment [5]. Further, the LI-RADS lexicon includes definitions and context of use for most of the terms that describe these LI-RADS M features [6, 7].

Despite the existence of LI-RADS M terms that may apply to CCA, there is a lack of standardized terminology specific for reporting CCA as an isolated finding, i.e., in patients that do not fit the LI-RADS population, as well as to describe additional imaging features specific to intra- or extrahepatic CCA that may further improve communication and patient care, both for clinical and research purposes. To address this gap, the Society of Abdominal Radiology (SAR) Disease Focused Panel (DFP) on CCA, developed a lexicon specific for CCA. This article will describe the process for developing the lexicon, introduce the lexicon terms, and provide a pictorial computed tomography (CT) and magnetic resonance imaging (MRI) atlas of the 15 vetted terms relating to the imaging findings of CCA.

## Lexicon development

The SAR DFP on CCA was established and had its first meeting at the SAR annual meeting in Austin TX in March 2023. One of the main priorities of the DFP was to establish a lexicon devoted to CCA to improve communication with clinicians and surgeons, and to supplement the LI-RADS lexicon with terms specific for CCA. In November 2023, an 11-member lexicon subgroup was formed (blinded for review). All 11 members of the lexicon group are radiologists with 1 to over 20 years of experience in abdominal imaging. Upon review of the literature and expert discussion and consensus, this same group identified 15 terms that were not already contemplated by the LI-RADS lexicon. Once the terms were agreed upon by the lexicon group, definitions, applicable modalities, comments related to the term, common synonyms, and type of CCA for which the term applies to were defined in an iterative fashion until all 15 terms and their associated definitions and comments were approved by the 11-member lexicon group.

On February 20, 2024, the proposed lexicon was sent to the entire 49-member roster of the SAR CCA DFP for review. Suggestions were taken and edits were made to the initially proposed lexicon until all members had a chance to give input on the lexicon. On April 28, 2024, the lexicon was put to vote by entire SAR CCA DFP. The goal was for 100% participation and each term needed over 90% approval to be accepted by the DFP. The survey was active until June 13, 2024. 39 multidisciplinary DFP members, including 2 surgeons, 2 pathologists, a radiation oncologist, and an interventional radiologist, all took part in the survey and all 15 terms achieved over 90% approval by the DFP. Table 1 provides a list of all 15 approved terms, their definitions, applicable modalities, and applicable type of CCA.

**Table 1:**
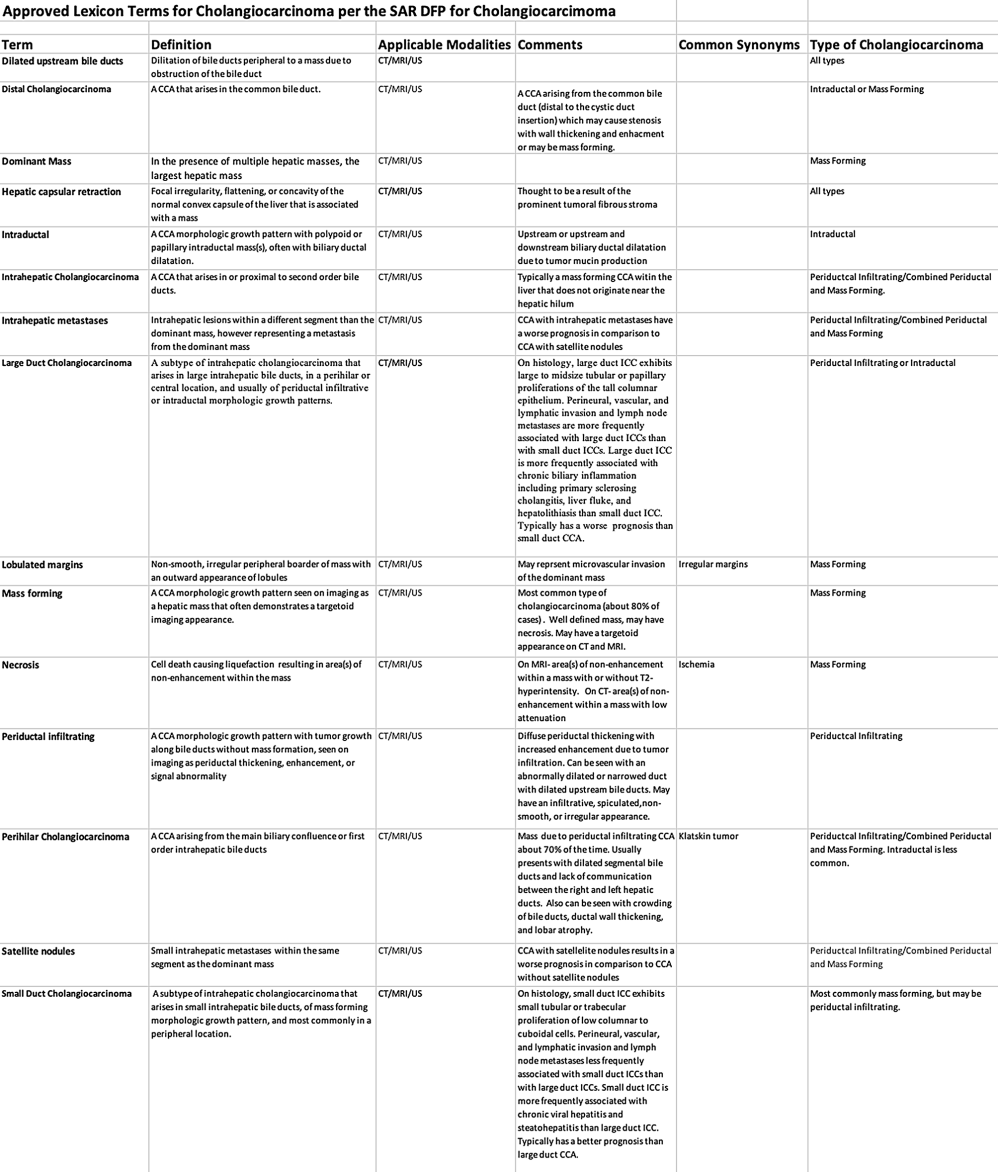
Approved lexicon terms for Cholangiocarcinoma per the SAR DFP for Cholangiocarcinoma

## Lexicon terms

### Intraductal

**Fig. 1:**
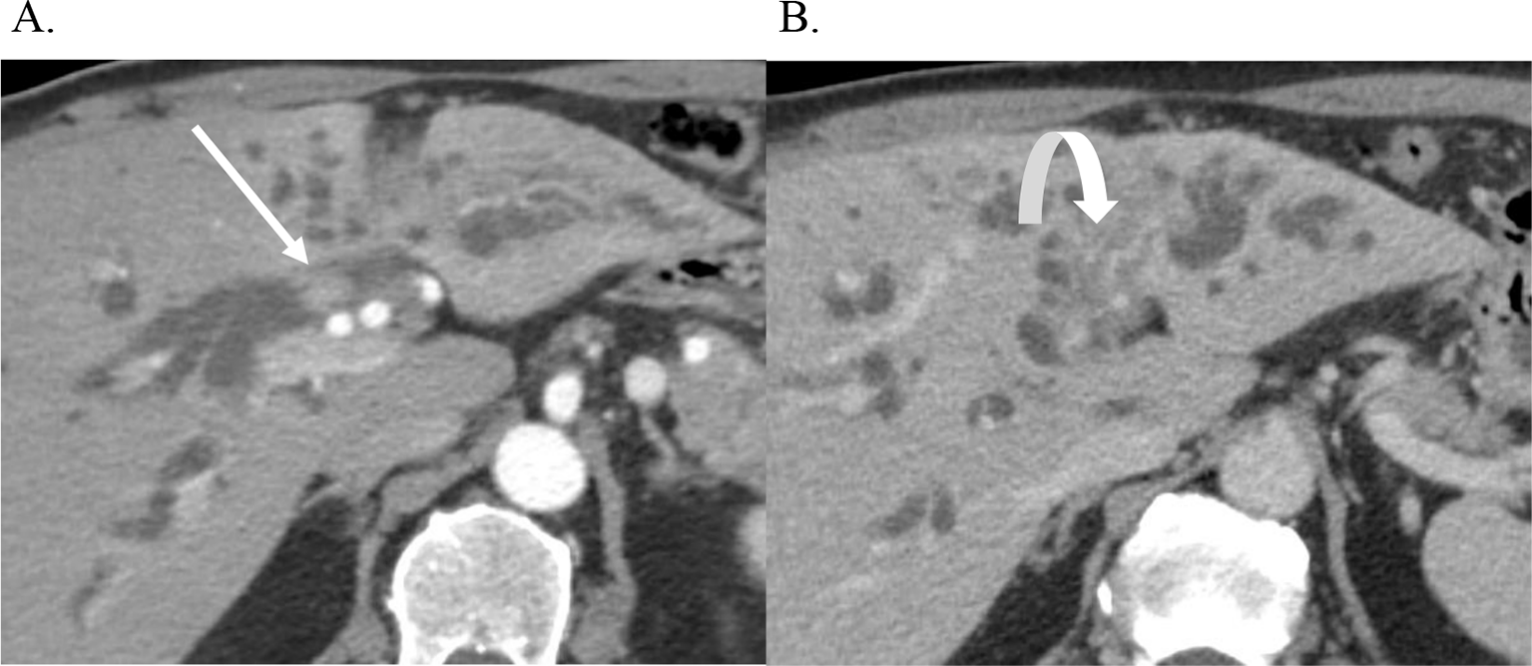
Intraductal CCA. Axial CT images with contrast demonstrate an intraductal perihilar mass (arrow) with upstream intrahepatic ductal dilatation (**A**). There is tumor thrombus in the left portal vein (curved arrow) (**B**)

An intraductal CCA is a morphologic growth pattern with a polypoid or papillary intraductal mass, often with biliary ductal dilatation (Fig. 1). There is typically upstream or both upstream and downstream biliary ductal dilatation due to either biliary obstruction and tumor mucin production [8]. According to the 5th edition of the World Health Organization (WHO), this morphologic growth pattern is considered a malignant transformation of intraductal papillary neoplasm of the bile duct (IPNB) [9]. Differentiating IPNB with intraductal CCA is difficult as they may appear similar on imaging. One differentiating feature of IPNB is that it lacks delayed enhancement compared to intraductal CCA due to the absence of fibrous tissue in IPNB [10, 11].

### Mass forming

**Fig. 2:**
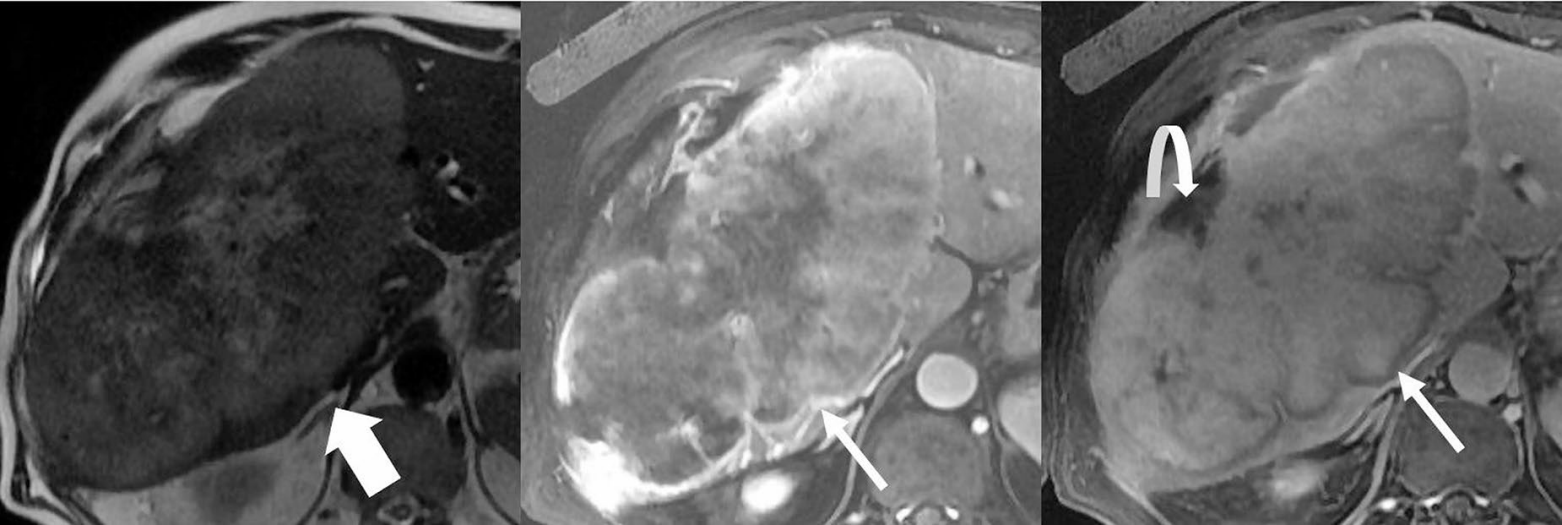
Mass Forming CCA. Axial T2 weighted image (**A**), and post-contrast T1 weighted arterial phase (**B**) and delayed phase (**C**) images demonstrate a large intrahepatic mass forming CCA that is mildly T2 bright (thick arrow, A), has rim APHE (arrow B) and delayed central enhancement and peripheral washout (thin arrow, C). There is associated capsular retraction (curved arrow)

A mass forming CCA is a morphologic growth pattern seen on imaging as a space occupying hepatic mass that often demonstrates a targetoid imaging appearance, as defined by the LI-RADS lexicon, i.e., target-like morphology on CT or MRI where the center and periphery of a mass have different imaging characteristics (Fig. 2) [6]. According to LI-RADS, the targetoid features of LR-M, which include a mass forming CCA in its differential include rim APHE, peripheral washout, delayed central enhancement, or a targetoid appearance in diffusion restriction, transitional phase, or hepatobiliary phase [7] and may have central necrosis [12]. Mass forming is most common growth-type of CCA comprising of about 80% of the CCA cases [12].

### Periductal infiltrating

**Fig. 3:**
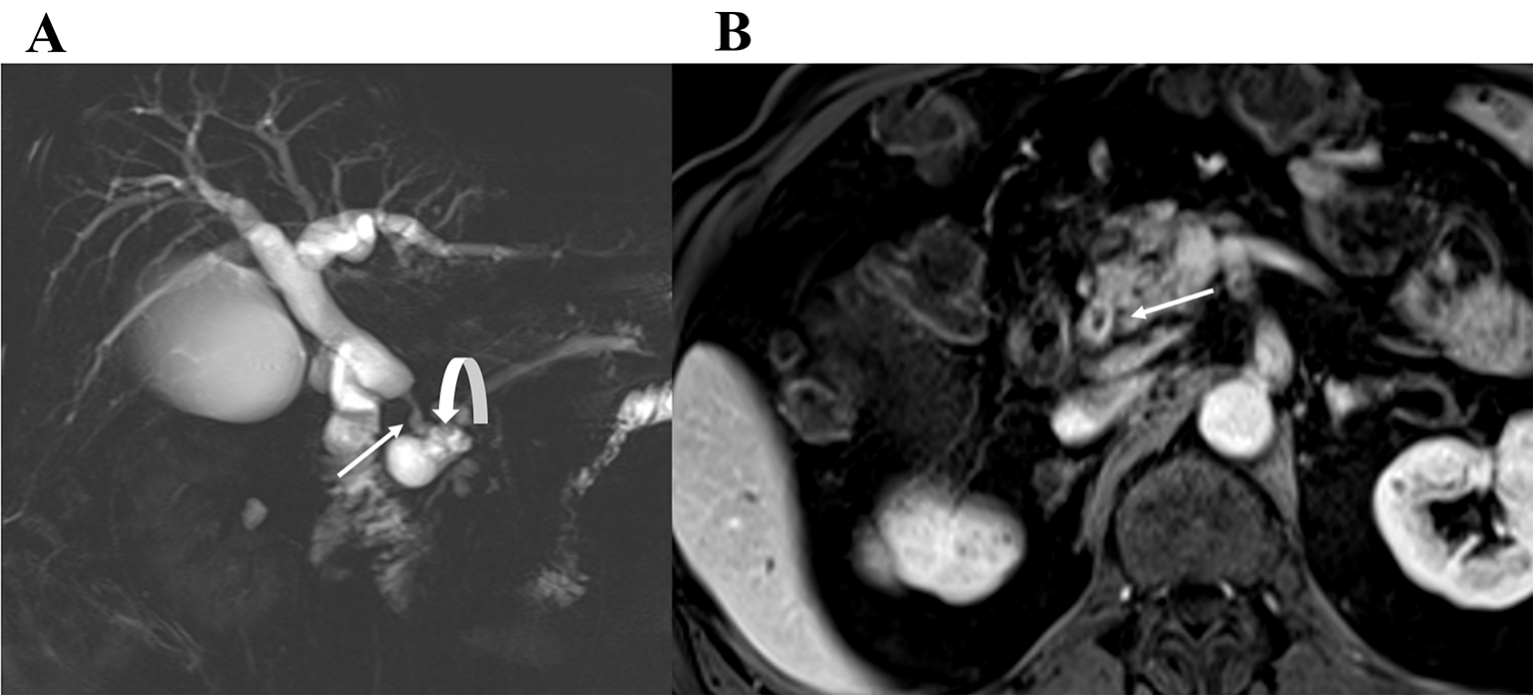
Periductal Infiltrating Cholangiocarcinoma.Coronal magnetic resonance cholangiopancreatography (MRCP) demonstrates a stricture of the common bile duct (CBD) (**A**). Incidentally noted is a 2 cm side branch intraductal papillary mucinous neoplasm in the pancreatic head (curved arrow). Post contrast T1 weighted image demonstrates narrowing of the CBD with wall thickening and enhancement (**B**)

A periductal infiltrating CCA is a morphologic growth pattern with tumor growth along the bile duct wall without mass formation, seen on imaging as periductal thickening, enhancement, or signal abnormality (Fig. 3). It can be seen with an abnormally dilated or narrowed duct with dilated upstream bile ducts. It may have an infiltrative, spiculated, non-smooth, or irregular appearance.

### Intrahepatic cholangiocarcinoma

**Fig. 4:**
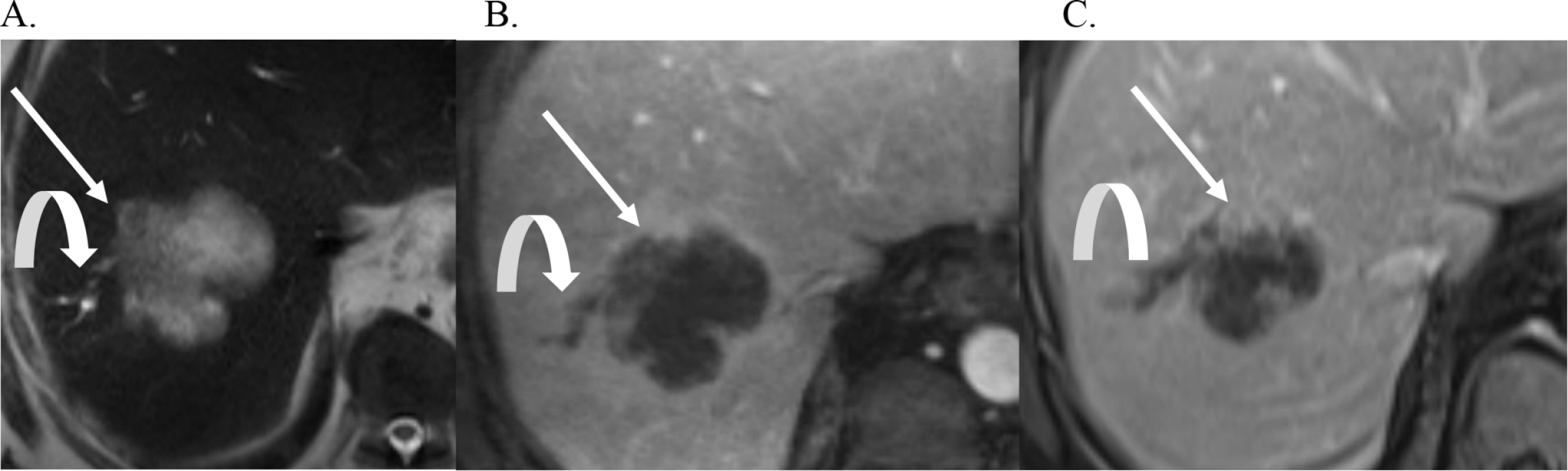
Intrahepatic Cholangiocarcinoma. Axial T2 (**A**), and post contrast T1-weighted portal venous phase (**B**) and delayed phase (**C**) images demonstrate a 6.1 cm mass forming CCA (arrow) in hepatic segment 7. There is rim enhancement on the portal venous phase (**B**) with centripetal central enhancement on the delayed phase (**C**). There are dilated upstream bile ducts peripheral to this mass (curved arrows)

Intrahepatic CCA arises within and/or proximal to second order bile ducts. This is typically an intrahepatic mass forming CCA (Fig. 4). Although approximately 80% of intrahepatic CCAs are of the mass forming type, other types of CCA’s may also be intrahepatic, such as periductal infiltrating and combined periductal/mass forming [13].

### Distal cholangiocarcinoma

**Fig. 5:**
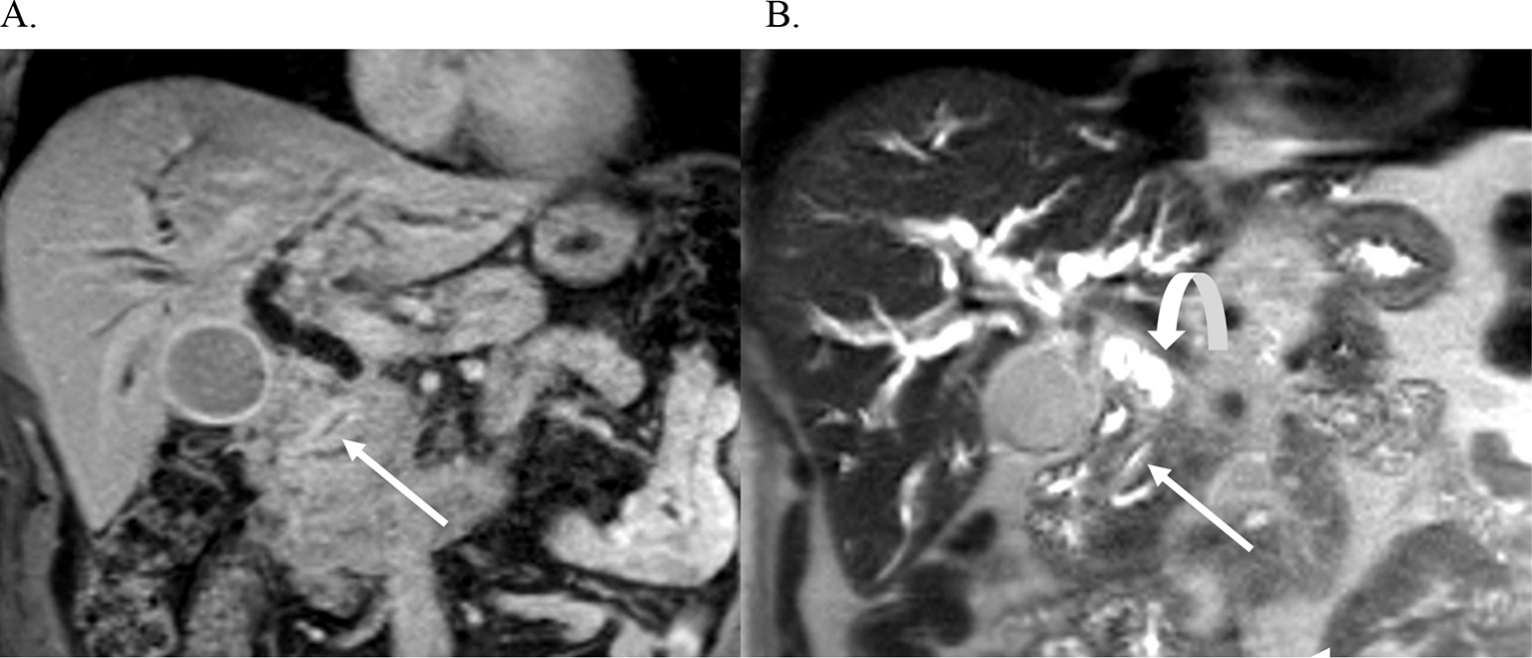
Distal Cholangiocarcinoma. Coronal contrast enhanced T1 weighted image (**A**) and coronal T2 weighted image (**B**) demonstrates a stricture in the distal CBD with wall thickening and enhancement (arrow) (**A**), and a stricture seen in the distal CBD (arrow) in **B**, causing dilatation of the upstream common bile duct and intrahepatic bile ducts (curved arrow)

A distal CCA is a tumor that arises in the CBD i.e., distal to the cystic duct insertion, which may cause stenosis of the duct, either as periductal infiltrating or mass forming (Fig. 5) [14]. Patients with distal CCA usually have a poor prognosis with approximately 50% of patients experiencing recurrence at 5 years after surgical resection [15].

### Perihilar cholangiocarcinoma

**Fig. 6:**
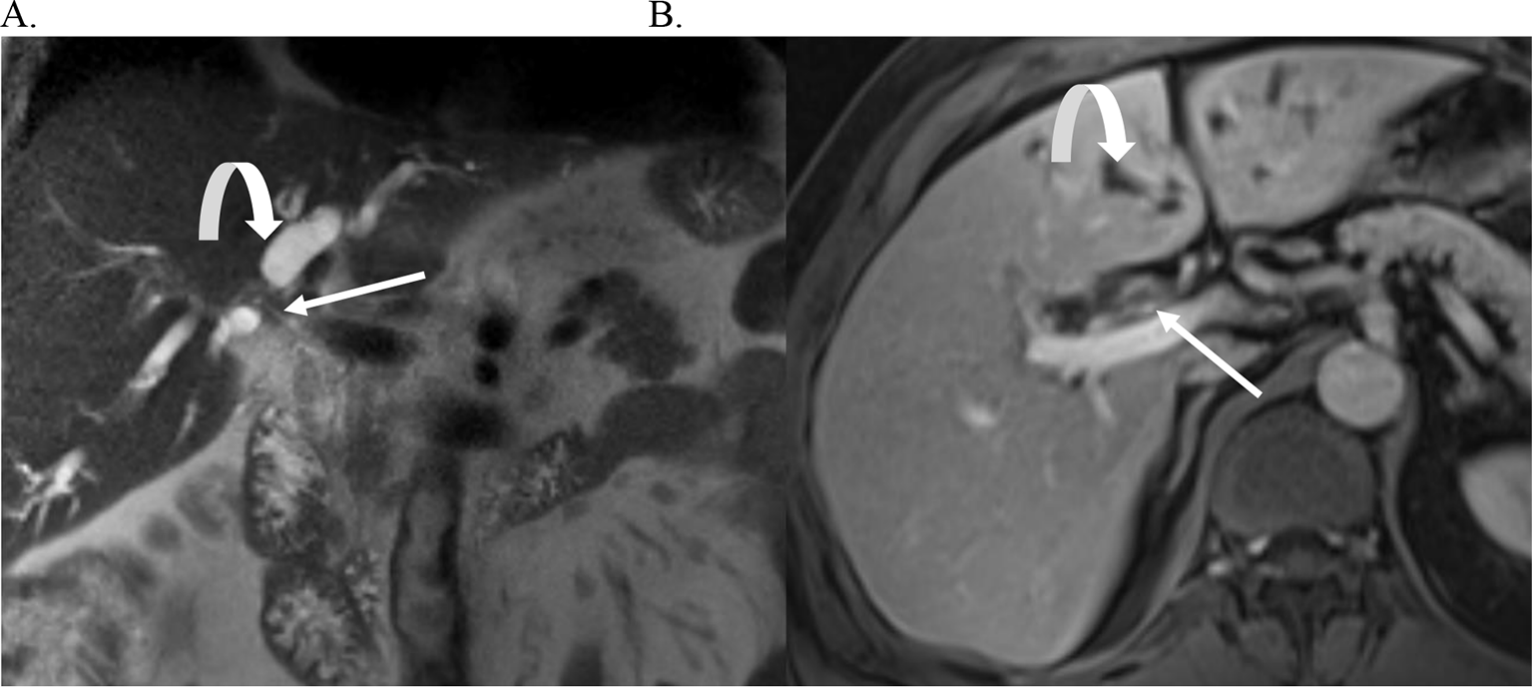
Perihilar Cholangiocarcinoma. Coronal T2-weighted image demonstrates a T2 dark stricture at the biliary hilum (arrow) (**A**). T1-weighted portal venous phase image demonstrates narrowing and wall enhancement of the common hepatic duct at the biliary hilum consistent with a periductal infiltrating perihilar CCA. There is upstream biliary ductal dilatation (curved arrows in **A** and **B**)

A perihilar CCA, also known as a Klatskin tumor, is a CCA arising from the common hepatic duct, main biliary confluence or first order intrahepatic bile ducts (Fig. 6). It is periductal infiltrating CCA approximately 70% of the time and usually presents with dilated segmental bile ducts lacking communication between the right and left hepatic ducts [16]. Additional imaging features include crowding of the bile ducts, ductal wall thickening, and lobar atrophy. Although it may have a mixed mass forming/periductal infiltrating morphology, this is less common than the periductal infiltrating morphology [17].

### Large duct cholangiocarcinoma

**Fig. 7:**
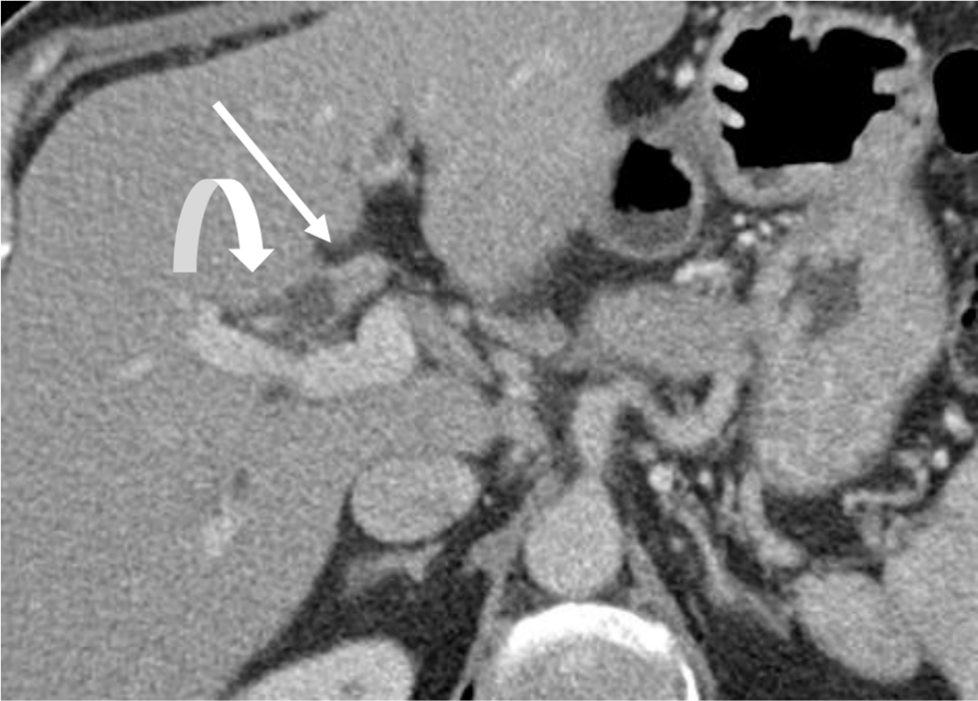
Large Duct Cholangiocarcinoma. Axial CT images with contrast demonstrate a periductal infiltrative mass (arrow), with wall thickening and enhancement of the common hepatic duct with dilated upstream bile ducts (curved arrow)

A large duct CCA is a subtype of intrahepatic CCA that arises in large intrahepatic bile ducts, in a perihilar or central location, and is usually of periductal infiltrative or intraductal in its morphologic growth pattern (Fig. 7). Large intrahepatic ducts consist of the first and second branches of hepatic bile ducts and contain peribiliary glands within the duct walls [18]. On histology, large duct CCA exhibits large to midsize tubular or papillary proliferations of the tall columnar epithelium that produce mucin [19]. Perineural, vascular, and lymphatic invasion and lymph node metastases are more frequently associated with large duct CCAs than with small duct CCAs [19]. Large duct CCA is more frequently associated with chronic biliary inflammation including primary sclerosing cholangitis, liver fluke infection, and hepatolithiasis and typically has a worse prognosis than small duct CCA [9]. On imaging, large duct CCA more frequently presents with poorly defined margins, vascular invasion, and lack of arterial hyperenhancement [20].

### Small duct cholangiocarcinoma

**Fig. 8:**
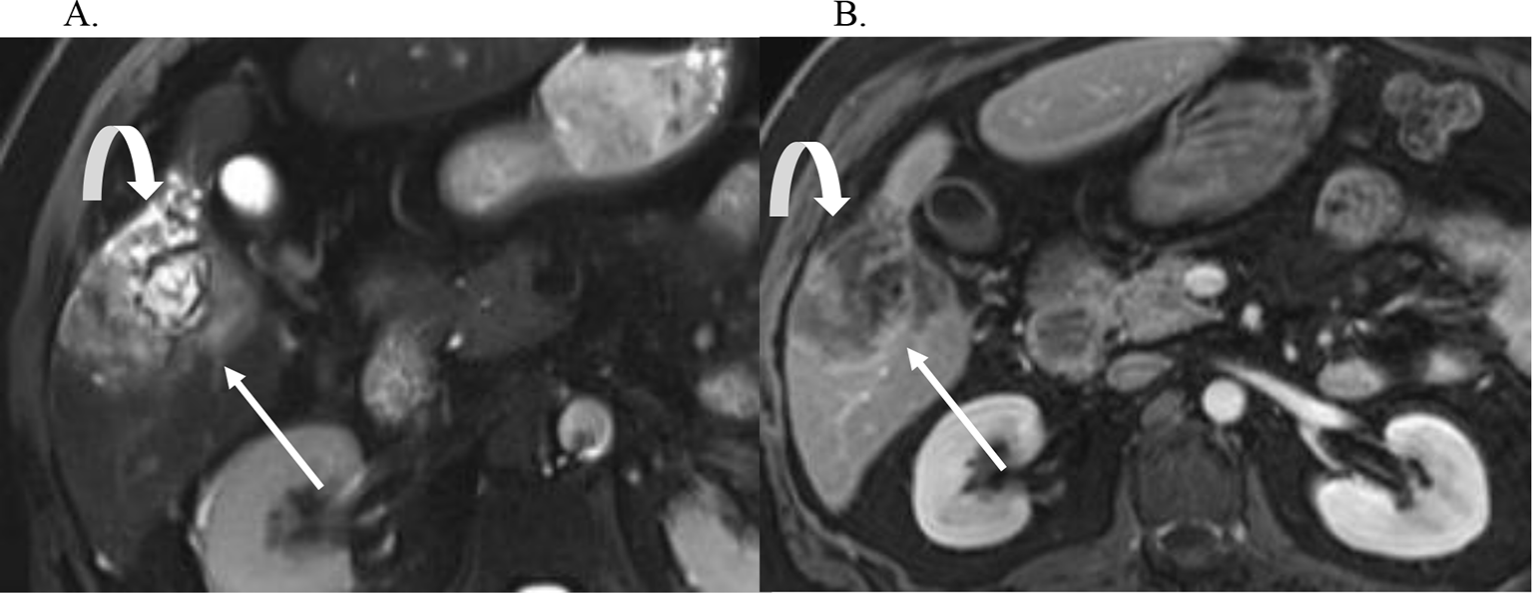
Small Duct Cholangiocarcinoma. Axial T2-weighted fat saturated image (**A**) and post-contrast image (**B**) demonstrate a T2 mildly bright mass in segment 5 of the liver (arrow) with rim enhancement and delayed central enhancement (arrow, **B**), consistent with a mass forming small duct CCA with associated capsular retraction (curved arrow)

A small duct CCA is a subtype of intrahepatic CCA that arises in small intrahepatic bile ducts, usually proximal to second order ducts and consisting of septal and interlobular bile ducts without peribiliary glands [18]. It is most commonly of mass-forming morphology and is commonly in a peripheral location in the liver (Fig. 8). It is composed of non-mucin-producing low columnar to cuboidal cells [9]. Perineural, vascular, and lymphatic invasion and lymph node metastases are less frequently associated with small duct CCAs than with large duct CCAs [19]. Small duct CCA is more frequently associated with chronic viral hepatitis and steatohepatitis and has a better prognosis than large duct CCA [19, 21]. On imaging, it is more frequently associated with rim APHE and a targetoid appearance on T2-weighted imaging, DWI, and the HBP [20].

### Dominant mass

**Fig. 9:**
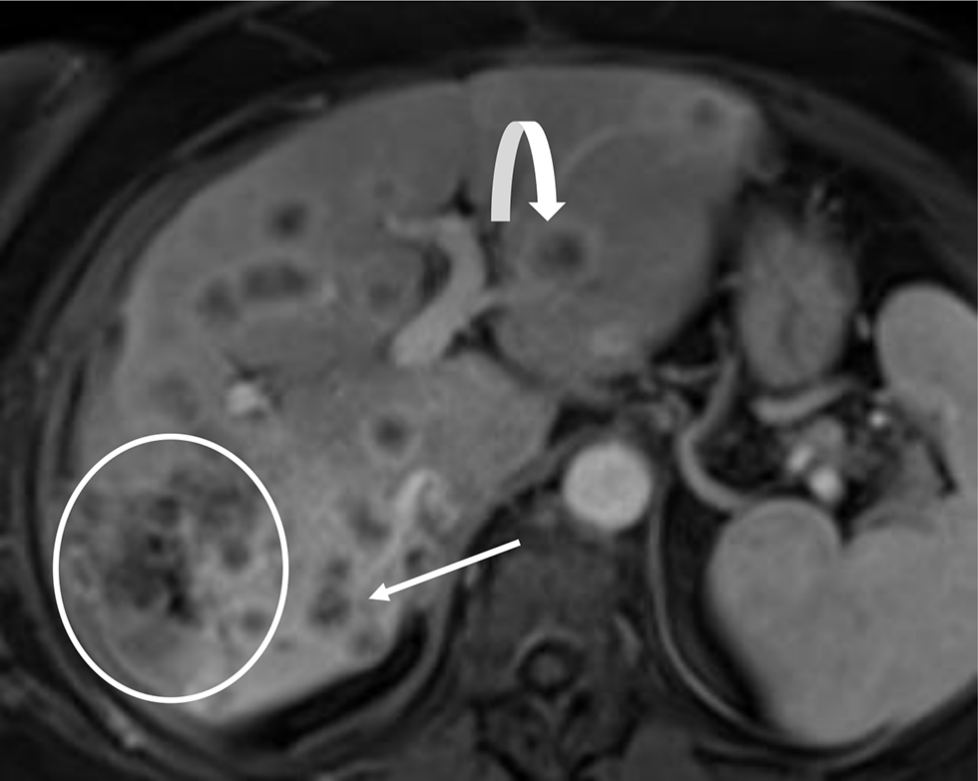
Dominant Mass, Intrahepatic Metastases, and Satellite Nodules. Axial post contrast T1-weighted MRI demonstrates a dominant mass forming CCA in segment 6 (white region of interest), regional segment 6 satellite nodules (thin arrow), and intrahepatic metastases in the left hepatic lobe (curved arrow)

The dominant mass is the largest intrahepatic mass forming CCA in the presence of multiple hepatic masses (Fig. 9).

### Intrahepatic metastases

In the presence of a dominant mass, smaller intrahepatic lesions within a different segment(s) are considered intrahepatic metastases (Fig. 9). CCA with intrahepatic metastases have a worse prognosis in comparison to CCA with satellite nodules [22].

### Satellite nodules

Satellite nodules are smaller intrahepatic lesions within the same segment as the dominant mass (Fig. 9) [23, 24]. CCA with satellite nodules results in a worse prognosis in comparison to CCA without satellite nodules [22].

### Dilated upstream bile ducts

**Fig. 10:**
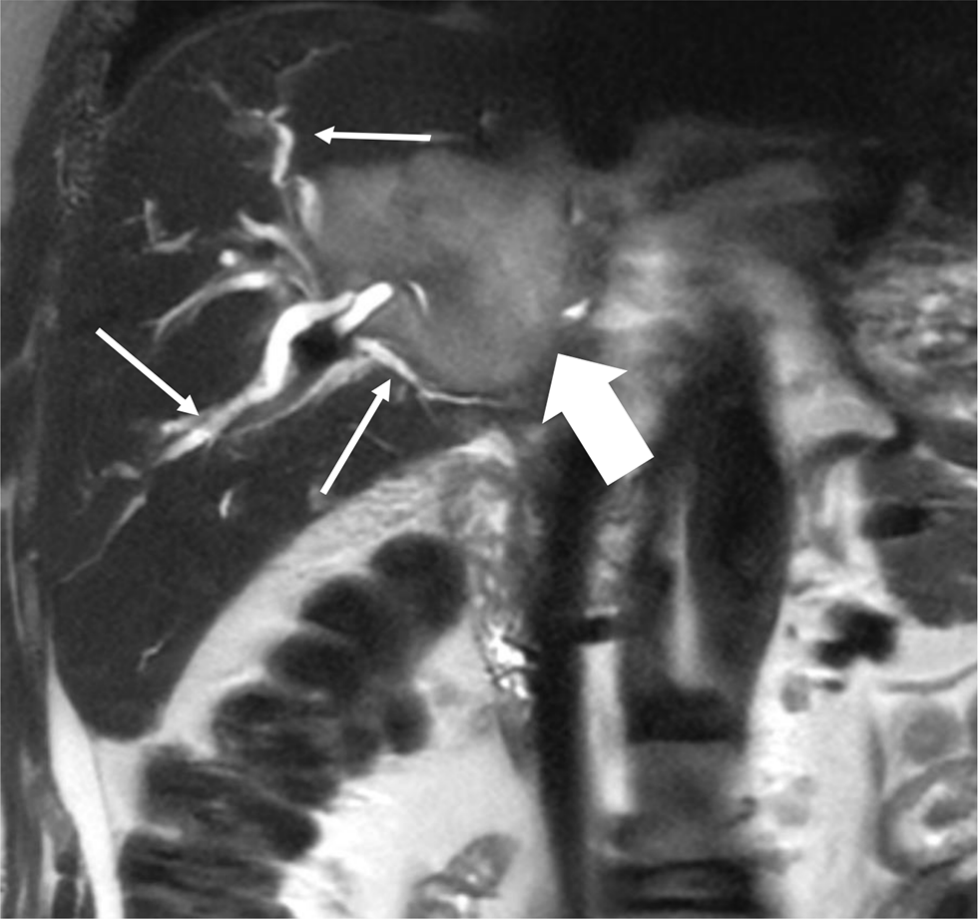
Dilated Upstream Bile Ducts. Coronal T2-weighted image demonstrates a large mildly T2 bright mass forming CCA in the right hepatic lobe (block arrow) causing dilated upstream bile ducts (arrows)

Dilated upstream bile ducts refers to dilatation of bile ducts peripheral to a CCA due to obstruction of the bile duct. This is seen almost with all morphological types of CCA (see Fig. 10).

### Hepatic capsular retraction

**Fig. 11:**
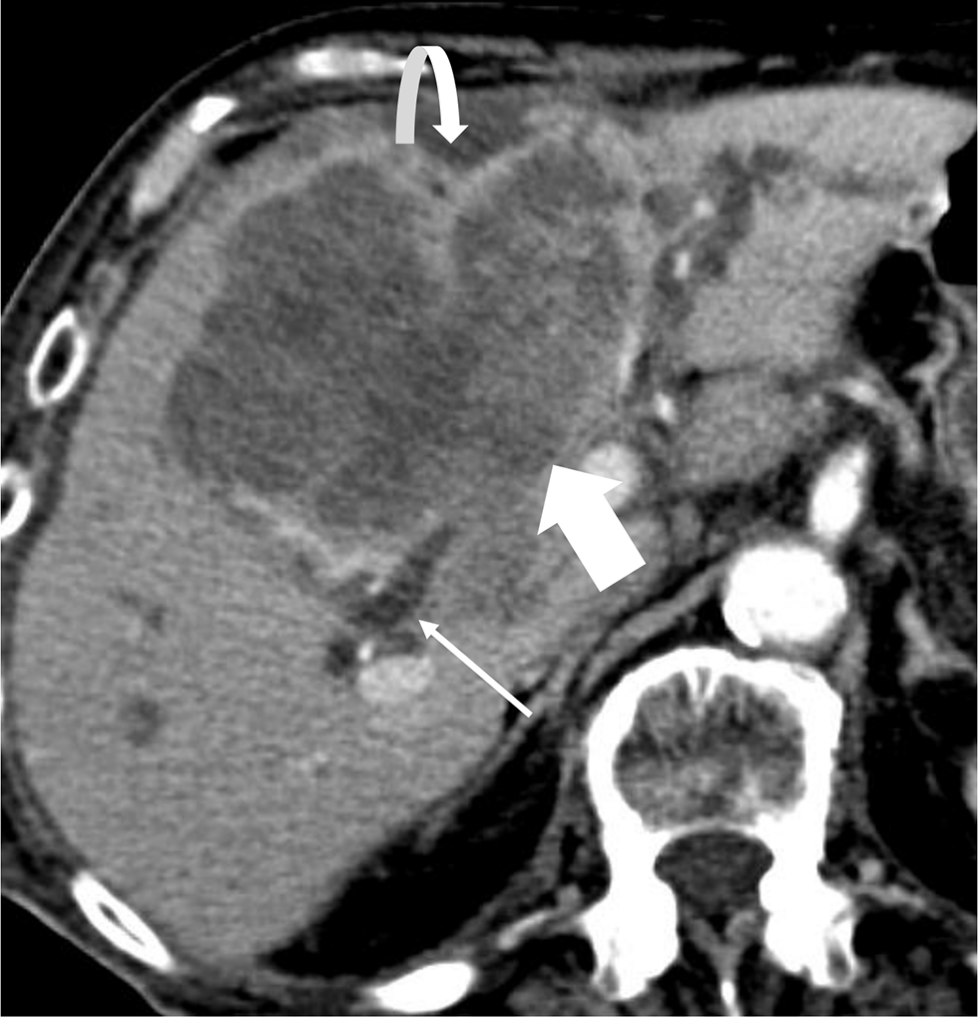
Hepatic Capsular Retraction. Axial contrast-enhanced CT demonstrates a large central mass forming CCA (block arrow) with hepatic capsular retraction (curved arrow) along the capsule of the liver. Also noted are dilated upstream bile ducts (thin arrow)

Hepatic capsular retraction refers to focal irregularity, flattening, or concavity of the normal convex capsule of the liver that is associated most often with mass forming CCA (Fig. 11). It is thought to be a result of desmoplastic reaction and prominent tumoral fibrous stroma [25].

### Lobulated margins

**Fig. 12:**
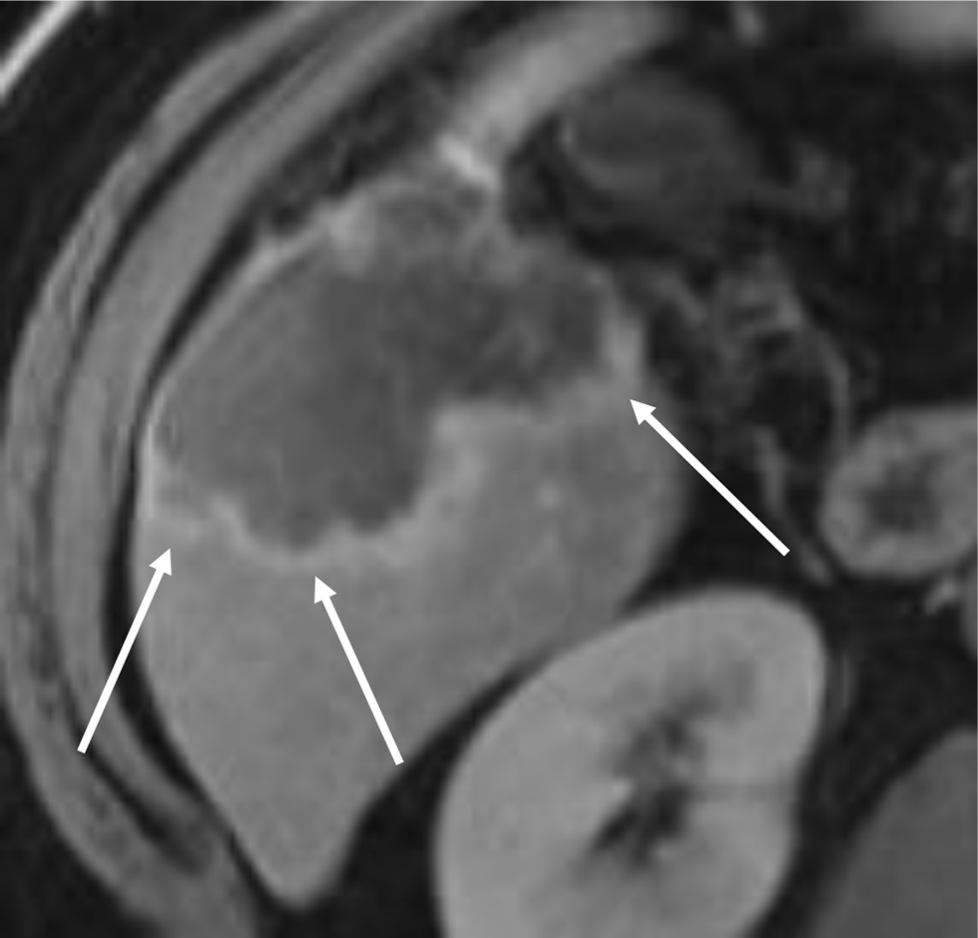
Lobulated Margins. Axial T1-weighted MRI in an early delayed phase demonstrates a peripheral mass forming CCA in the right hepatic lobe with lobulated margins (arrows) and rim enhancement

Lobulated margins refer to a non-smooth or irregular peripheral border of a mass with an outward appearance of lobules associated with mass forming CCA (see Fig. 12). Lobulated margins may represent microvascular invasion of the dominant mass [13].

### Necrosis

**Fig. 13:**
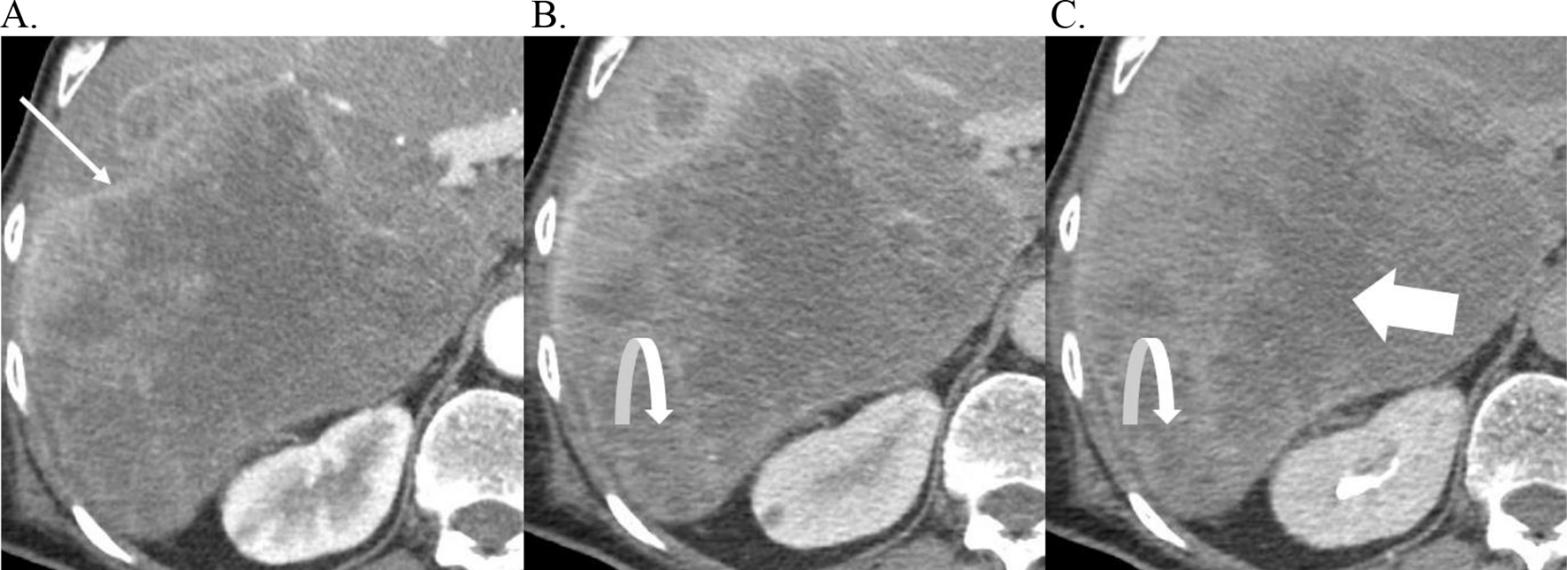
Necrosis. Axial contrast enhanced CT in the arterial phase (**A**), portal venous phase (**B**), and delayed phase (**C**), demonstrating a pathologically proven necrotic CCA with rim APHE (arrow), delayed central enhancement (curved arrow), and non-enhancing necrosis centrally (large arrow)

Necrosis refers to cell death causing liquification resulting in areas of non-enhancement with a mass forming CCA. On MRI, necrosis appears as areas of non-enhancement within a mass with or without T2-hyperintensity. On CT, it appears as areas of non-enhancement within a mass with low attenuation. Although the clinical implication of necrosis is not well studied, preliminary data suggests a favorable prognostic role of necroptosis in CCA (see Fig. 13) [26].

### Summary

The SAR DFP on CCA lexicon is intended to complement the LI-RADS lexicon by expounding on imaging terms and features specific for CCA which were not defined in the terms related to LI-RADS M. It is our goal for these terms to be used in clinical reports and scientific papers relating to CCA. As more data and terms become apparent that are directly related to CCA, this lexicon will grow and will be updated over time. Overall, adopting and using this lexicon, as with all imaging lexicons, will help facilitate clear and direct communication between radiologists and clinicians while also helping to standardize terminology for scientific research.

## Data Availability

No datasets were generated or analysed during the current study.
